# Comprehensive Quality Evaluation for Medicinal and Edible Ziziphi Spinosae Semen before and after Rancidity Based on Traditional Sensory, Physicochemical Characteristics, and Volatile Compounds

**DOI:** 10.3390/foods11152320

**Published:** 2022-08-03

**Authors:** Zhenying Liu, Liang Xu, Pingping Song, Cui Wu, Bo Xu, Zhuojun Li, Zhimao Chao

**Affiliations:** Institute of Chinese Materia Medica, China Academy of Chinese Medical Sciences, Beijing 100700, China; liuzy9607@163.com (Z.L.); xuliang9988@126.com (L.X.); songpingping122@163.com (P.S.); wucuidalian@163.com (C.W.); xubo_345@163.com (B.X.); 18811385399@163.com (Z.L.)

**Keywords:** medicinal and edible, Ziziphi Spinosae Semen, rancidity, traditional sensory, physicochemical characteristic, volatile compound

## Abstract

To comprehensively evaluate the quality of medicinal and edible Ziziphi Spinosae Semen (ZSS, the dried ripe seeds of *Ziziphus jujuba* var. *spinosa*) before and after rancidity during storage, some indicators including traditional sensory properties, physicochemical characteristics, and volatile compounds were analyzed. As a result, compared with the normal samples, the rancid samples of ZSS produced a darker color, a bitter taste, and an irritating odor, increased moisture content, electrical conductivity, fatty oil content, and acid value, and decreased water- and alcohol-soluble extract contents and pH value. Among them, the acid value had significant difference (*p* < 0.01) from 3.90 of normal ZSS to 18.68 mg/g of rancid ZSS. A total of 39 volatile compounds were identified in samples, including 20 in normal ZSS and 38 compounds in rancid ZSS. Nineteen common compounds were identified in normal and rancid samples. Among them, the content of 10 compounds such as *δ*-limonene, (*R*,*R*)-2,3-butanediol, and (*R*,*S*)-2,3-butanediol was decreased but that of nine compounds such as acetic acid, *n*-octanoic acid, and *n*-nonanoic acid was increased in rancid ZSS. Nineteen unique compounds such as *β*-phellandrene, *α*-pinene, and 3-carene were detected and only one compound, *δ*-cadinene, was not detected in rancid ZSS. In addition, eight short-chain organic acids, acetic, propanoic, butanoic, pentanoic, hexanoic, heptanoic, octanoic, and nonanoic acids, were new products in rancid ZSS, and it was speculated that the production of a series of organic acids might be the material basis of irritating odor after normal ZSS became rancid. This is the first report that a series of short-chain organic acids have been found in a rancid substance. In conclusion, there was a significant difference between normal and rancid ZSS. These indicators could be used as an early warning for judging the rancidity phenomenon of medicinal and edible ZSS. In addition, this is the first comprehensive evaluation about the rancidity process of a medicinal and edible substance.

## 1. Introduction

Ziziphi Spinosae Semen (ZSS), the dried ripe seeds of *Ziziphus jujuba* Mill. var. *spinosa* (Bunge) Hu ex H.F. Chou (Fam. Rhamnaceae), has been commonly used in Traditional Chinese Medicine (TCM) for the treatment of deficiency vexation, insomnia, fright palpitations, dream-disturbed sleep, and weak constitution [1]. In modern clinical practice, ZSS has many medicinal benefits such as treatment for calming, fright palpitations, dreaminess, and tranquilizing the nerves, and is most commonly used to treat insomnia and anxiety [2,3,4]. It has some pharmacological activities such as regulating immunity, ameliorating memory and learning, protecting the cardiovascular system, and hypnosis [5,6,7]. With the transformation in medical purposes and health perspectives, there are some changes that have taken place in people’s perception of TCM. People prefer to prevent disease through diet in their daily life, and as a result, healthy food with disease prevention has become increasingly popular [8]. ZSS, as a medical and edible substance, has been widely used in edible usages such as wine, juice, and syrup, and pushes forward an immense positive influence, gaining great popularity among consumers in the healthcare industry [9,10].

ZSS is abundant with fatty oil and prone to deterioration, especially during storage with inappropriate conditions such as high temperature and high humidity. After a long period of storage, ZSS produces an unpleasant smell, especially in summer which is hot and humid, which is the so-called rancidity phenomenon [11]. It is the result of some unsaturated fatty acids coming into contact with oxygen to bring out a series of peroxides. Then, these peroxides are degraded into a complex mixture of volatile compounds including aldehydes, ketones, terpenes, and acids. In addition, they are potentially detrimental for health since rancid oxidation products are implicated in the pathogenesis of some diseases. Therefore, it is necessary to carry out technical supervision on the quality of ZSS and to distinguish between normal and rancid samples. However, there are no reports about comprehensive evaluation including traditional sensory and physicochemical characteristics and volatile compounds before and after rancidity of medicinal and edible substances at present.

In this study, the traditional sensory properties including color, taste, and odor, physicochemical characteristics including moisture content, water and alcohol extract content, pH value, electrical conductivity, fatty oil content, and acid value, and volatile compounds were determined to comparatively evaluate the quality before and after rancidity of ZSS.

## 2. Materials and Methods

### 2.1. Materials

#### 2.1.1. Reagents

Hydrogen phthalate buffer was purchased from INESA Scientific Instrument Co., Ltd. (Shanghai, China), ether, 95% ethanol, and petroleum ether (60–90 °C) were purchased from Fuyu Fine Chemical Co., Ltd. (Tianjin, China), 1% phenolphthalein indicator was purchased from Reagecon Diagnostics Co., Ltd. (Clare, Ireland), 0.01 N NaOH ethanol solution was purchased from General Reagents Co., Ltd. (Shanghai, China). All of the chemicals and reagents used were of analytical-grade purity.

#### 2.1.2. Samples Collection

The normal ZSS samples without deterioration were purchased in August 2020 from Changlong Chinese Herbal Medicine Co., Ltd., Anguo City, Hebei Province, which were identified as the dried ripe seeds of *Ziziphus jujuba* Mill. var. *spinosa* (Bunge) Hu ex H. F. Chou (Family Rhamnaceae) by Prof. Zhimao Chao (Institute of Chinese Materia Medica, China Academy of Chinese Medical Sciences) according to the description in Flora of China (Editorial Board of Flora of China, 2012). The voucher specimens (No. ZSS20200815) were deposited at 1022 laboratory of the Institute of Chinese Materia Medica, China Academy of Chinese Medical Sciences, Beijing, China.

#### 2.1.3. Samples Preparation

**Figure 1:**
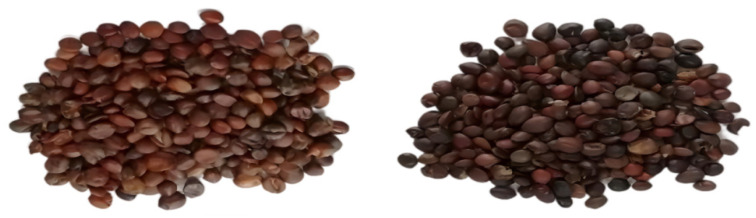
The normal (**left**) and rancid (**right**) ZSS samples.

The rancid ZSS samples were obtained by transferring normal ZSS samples to an HQ-250 incubator (Zensan Electrical Technology Co., Ltd., Shanghai, China) with temperature at 40 °C and relative humidity (RH) of 75% for 6 months. The normal and rancid samples are shown in Figure 1.

### 2.2. Methods

#### 2.2.1. Traditional Sensory Analysis

Normal and rancid ZSS samples were randomly selected, ground into powder, and dried at 40 °C for 3 h. The traditional sensory evaluation of color, taste, and odor was performed by five trained laboratory members from the Institute of Chinese Materia Medica, China Academy of Chinese Medical Sciences (Beijing, China).

#### 2.2.2. Physicochemical Characteristics Analysis

##### Moisture Content

The moisture content of ZSS samples was determined by the drying method as described in the General Rule 0832 of 2020 Chinese Pharmacopoeia [12].

##### Extracts Content

The ZSS samples were crushed until they could pass through the No.2 Taylor Standard Sieve. The content of water- and alcohol-soluble extracts was measured by the hot-dip method as described in General Rule 2201 of 2020 Chinese Pharmacopoeia [10] with water and 95% ethanol as solvents, respectively.

##### pH Value

Five grams of sample power and 100 mL freshly deionized water were placed in a beaker. Then, the mixture was homogenized and centrifuged at 3000 r/min at 25 °C for 5 min. The supernatant was used for pH determination by using a PHS-3E electronic pH meter (INESA Scientific Instrument Co., Ltd., Shanghai, China) with a potassium hydrogen phthalate buffer at pH 4.00.

##### Electrical Conductivity

Five grams of sample power and 100 mL freshly deionized water were placed in a beaker, homogenized and centrifuged at 3000 r/min for 5 min. Electrical conductivity of the supernatant was measured at 25 °C with a DDS-11A conductivity meter (INESA Scientific Instrument Co., Ltd., Shanghai, China).

##### Fatty Oil Content

Twenty grams of sample power and 150 mL petroleum ether (60–90 °C) were placed in a Soxhlet extraction apparatus under reflux for 6 h. The fatty oil content was measured by weighing again after the solvent was recovered.

##### Acid Value

The acid value was determined by dissolving the above fatty oil samples in an ethanol–ether mixture with the addition of 1% phenolphthalein indicator and by titrating with 0.01 N NaOH ethanol solution until a pinkish color appeared steadily, which was in accordance with the method in General Rule 0713 of 2020 Chinese Pharmacopoeia [12].

#### 2.2.3. Volatile Compound Analysis

The volatile compounds of normal and rancid samples were extracted using the headspace solid phase micro-extraction (HS-SPME) method. A 5.0 g sample was weighed accurately, placed in a 20 mL spiral headspace vial with a polytetrafluoroethylene (PTFE) septum, and equilibrated at 60 °C for 5 min. An SPME 65 μm carboxene/polydimethylsiloxane/divinylbenzene (CAR/PDMS/DVB) fiber (Supelco, Bellefonte, PA, USA) was extended through the needle and exposed to the headspace of the vial to adsorb volatile compounds at 60 °C for 30 min. Then, the needle was immediately injected into the GC injection port at 250 °C for 2 min to desorb volatile compounds.

The volatile compounds from the samples were subsequently analyzed by gas chromatography–mass spectrometry (GC-MS) according to the following conditions. For chromatography conditions, a GC-MS QP2010 plus HP-5MS (Shimadzu, Tokyo, Japan) was used with INNO WAX polyethylene glycol capillary column (30 m × 0.25 mm × 0.25 μm) (Agilent, Palo Alto, CA, USA). The splitless injection mode was used. High-purity helium, as a carrier gas, was used with a constant flow rate of 1 mL/min. The heating program was as follows: the initial temperature was 50 °C and held for 3 min, increased to 160 °C at a rate of 30 °C/min for 3 min, increased to 230 °C at a rate of 10 °C/min and held for 2 min. For MS conditions, the ion source temperature was 200 °C, the interface temperature was 250 °C, the solvent delay time was 1 min, and the scanning range was *m*/*z* 50 to 400. The mass spectra were obtained at 70 eV with electron ionization (EI) mode.

The mass spectral primary identification of volatile compounds in the samples was carried out by comparing with the National Institute of Standards and Technology (Gaithersburg, MD, USA) (NIST) 11. The qualitative analysis of mass spectral data was verified by comparing the retention time and mass spectra of identified compounds. The quantitative analysis of each compound was determined by peak area normalization of total ion chromatography.

### 2.3. Statistical Analysis

Samples were analyzed three times in triplicate experiments, and the results were expressed as mean value ± standard deviation (SD). Significance was calculated by one-way analysis of variance (ANOVA) and Student’s *t*-test, followed by Tukey’s multiple comparison test (*p* < 0.05) in the GraphPad Prism 9 software (San Diego, CA, USA). Pearson’s correlation coefficients were calculated by using SPSS 26.0 software (Chicago, IL, USA). Origin software (OriginLab Corporation, Northampton, MA, USA) was used for data analysis.

## 3. Results and Discussion

### 3.1. Traditional Sensory Analysis

Sensory indicators of color, taste, and odor are the key evaluation indexes of TCMs, which are considered to be important components and intuitive attributes in the identification of traits [13,14,15]. Ben Cao Yuan Shi, the book of Origins of the Materia Medica, emphasized the significance of color, taste, and odor as identifiers of high-quality medicinal herbs [16].

There is a significant difference between different qualities of TCM samples. For color, a fresh and normal Lycii fructus sample is true red and that of a poor and deteriorated sample is dark red; the cross-section of Scutellariae radix is yellow and an abnormal one is green; and the normal color of Armeniaceae semen amarum is pale yellow and an abnormal one is bright or dark yellow. For taste, the normal Piperis kadsurae caulis is bitter and spicy; the normal Sinomenii caulis is not spicy; and the natural Bovis calculus is bitter first and then sweet with a cool feeling and no fishy smell. For odor, the normal Ferulae resina has a particularly foul smell, Moschus has a particularly fragrant smell, and Ginseng radix and rhizoma have a slight fragrant. These significant characteristics are regarded as an important basis of initial assessment of quality.

In this study, the normal and rancid ZSS samples were subject to traditional sensory analysis by artificial methods including seeing, tasting, and smelling. The results of color, taste, and odor are shown in Table 1. Compared with the normal samples, it was found that the color became darker, the taste became bitter and sourer, and the odor became irritating and unpleasant for rancid samples, which were the results of changes in the properties of ZSS itself. In detail, the degree of rancidity of fatty oils is accelerated due to unfavorable conditions such as high temperature, humidity, and oxygen concentration. More peroxides and free fatty acids are produced in whole process, which continue to decompose into some aldehydes and ketones with short carbon chains. Then, these chemical changes result in ZSS developing a special pungent odor and darker color.

During the storage link of ZSS, the rancidity phenomenon results in a series of changes in chemical components, especially volatile compounds that have a unique odor and smell, including olefins, terpenes, aldehydes, ketones, and acids. These changes are finally reflected in the color, taste, and odor. Consumers can identify the quality according to these characteristics, eliminate rancid samples, and pick high-quality samples.

### 3.2. Physicochemical Characteristic Analysis

The physicochemical characteristics, such as moisture content and acid value, of the rancid ZSS samples were also changed significantly, and the results are shown in Table 2.

Moisture content plays an important role in reflecting the quality and stability of foods and TCMs [17,18]. Storage conditions with high moisture content are beneficial for the growth of microbes, leading to the production of more free fatty acids and even unpleasant odor [19,20,21]. Compared with normal ZSS, rancid samples had been placed in a high-temperature and -humidity condition, resulting in a higher moisture content, and became typical unqualified samples.

Water- and alcohol-soluble extracts are important indicators for judging the quality of food and TCMs. For example, water- and alcohol-soluble extracts of *Ganoderma lucidum* have direct lifespan elongation effects and potential anti-aging properties [22]. Alcohol−soluble extracts of *Urtica hyperborea* showed remarkable activity in reducing uric acid [23]. The content of water- and alcohol-soluble extracts in rancid samples was lower than that in normal samples, which was also an important reflection of the deterioration of the quality of ZSS.

The pH value is one of the indicators to be considered for foods or TCMs, as the potential influence of pH value on durability and shelf life is an important factor for their quality. The pH value could affect the biological tolerance and stability of formulations [24]. The pH of TCMs could limit the growth of microorganisms, contributing to the stability [25,26]. For ZSS samples, the pH value of rancid samples was slightly lower than that of normal samples. There was no significant difference between normal and rancid samples.

The electrical conductivity can indicate changes in the content of ionic substances such as some ions, proteins, and organic acids [27,28,29]. ZSS is rich in fatty oils, and the content of these substances that cause change in electrical conductivity was low, so there was no significant change.

Fatty oils are widely distributed in seed TCMs. Many studies suggested that fatty acids were important bio-compounds which took part in complex metabolic pathways [30]. After ZSS became rancid, the content of the fatty oils obtained by the extraction of petroleum ether showed a significant decrease, which indicated that fat-soluble fatty oils were decomposed in high-temperature and -humidity conditions. This was a common deterioration phenomenon of seed TCMs rich in fatty oils. Related studies found that the content of fatty oils decreased in almond and walnut after rancidity during storage link [31,32].

The acid value is one of the important safety indicators of seed TCMs. The 2020 edition of the Chinese Pharmacopoeia has established limit standards for the acid value of some TCMs that are prone to rancidity [1]. When the acid value is higher than the limit value, it means that the TCM has deteriorated. A related study suggested that the acid value of Fructus Trichosanthis was significantly increased after the deterioration [33]. The acid value of ZSS in this study quickly reached 18.68 after rancidity, which was consistent with the result of the related reference, indicating that acid value was a key indicator of significant (*p* < 0.05) changes in the rancidity process.

### 3.3. Volatile Compound Analysis

The detection of volatile compounds is an important method for evaluating the quality of foods and TCMs during storage link. For example, the content of esters generally decreased and the content of aldehydes, ketones, alcohols, acids, and pyrazines generally increased during storage link [34,35,36,37]. Therefore, it is necessary to explore the changes in volatile compounds before and after rancidity of ZSS samples.

**Figure 2:**
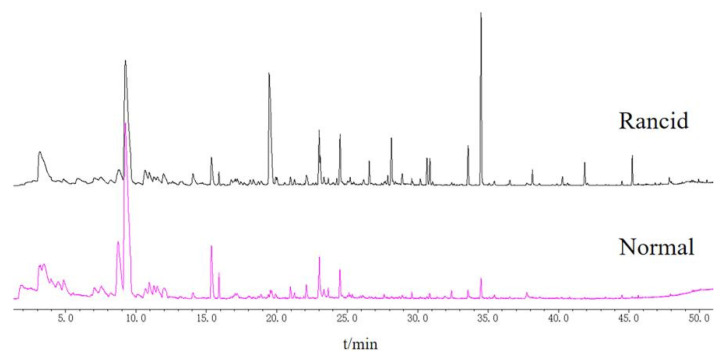
GC-MS total ion chromatogram of volatile components from normal and rancid ZSS samples.

In this study, HS-SPME technology was used to extract volatile components, and GC-MS technology was used to separate and identify volatile compounds. A total ion chromatogram is shown in Figure 2. The result showed that there was a significant difference between normal and rancid samples regarding their volatile components.

The results of volatile compounds are shown in Table 3. These identified compounds showed that mass spectrum and retention times were identical to standards under the given chromatographic/mass spectrometric conditions. For example, compound 2 (3.46 min) yielded a parent ion at *m*/*z* 170 and fragment ions at *m*/*z* 155, 112, 99, and 57, and was identified as 2,2,4,6,6-pentamethylheptane according to the NIST database. Compound 14 (19.48 min) displayed a parent ion at *m*/*z* 60 and fragment ions at *m*/*z* 45, 43, 29, and 15, and was identified as acetic acid. Compound 21 (24.48 min) produced a parent ion at *m*/*z* 90 and fragment ions at *m*/*z* 75, 57, 45, and 29, and was identified as (*R*,*S*)-2,3-butanediol.

**Figure 3:**
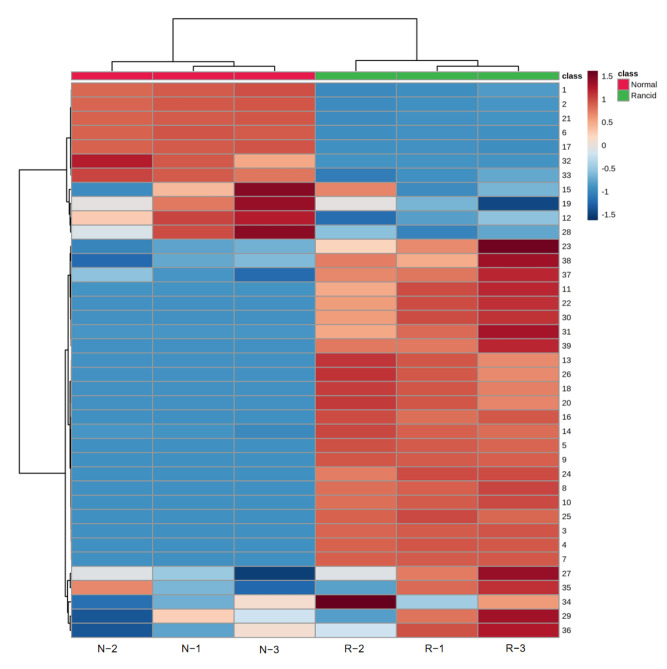
Heat-map and HCA of volatile compounds in normal and rancid samples. N−1, N−2, and N−3 represent normal samples. R−1, R−2, and R−3 represent rancid samples. The numbers on the right represent the compound number of Table 3. The deeper the red, the higher the content; the deeper the blue, the lower the content of the compound in the samples.

As a result, 39 volatile compounds were identified from the normal and rancid ZSS, including 20 in normal and 38 in rancid samples, and covering acids, alcohols, alkenes, aldehydes, ketones, etc. Then, they were selected and used to plot a heat-map and hierarchical cluster analysis (HCA) to find relatively homogeneous clusters of samples and to illustrate the content changes in volatile compounds from normal and rancid samples. As shown in Figure 3, normal and rancid samples were divided into two groups under HCA, and the different content levels of these compounds in the normal and rancid samples could be intuitively and clearly observed, suggesting that there was a significant difference between normal and rancid samples and enabling further analysis of common and unique compounds.

**Figure 4:**
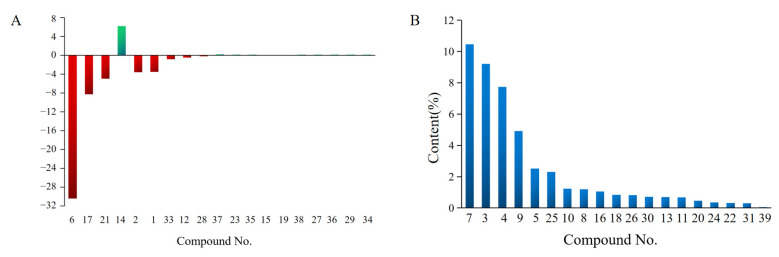
Increase (green) and decrease (red) in common volatile compounds after rancidity (**A**). The content of unique volatile compounds in rancid samples (**B**).

Both normal and rancid samples had 19 common compounds. As shown in Figure 4A, the content of 10 compounds was decreased but that of nine compounds was increased in rancid samples. For 10 decreased compounds, the relative content of *δ*-limonene had the largest decrease from 46.43 to 15.97%, followed by (*R*,*R*)-2,3-butanediol from 13.22 to 4.92%, and (*R*,*S*)-2,3-butanediol from 10.40 to 5.40%. *δ*-Limonene, a principal active compound of limonenes, was frequently used as a dietary supplement and fragrance ingredient for cosmetics products [38,39]. The largest decrease in *δ*-limonene content resulted in a weaker aromatic odor from rancid samples, which was consistent with the result that the content of limonenes decreased with the prolongation of storage time and affected the flavor in fried pepper (*Zanthoxylum bungeanum*) oil [40]. Related studies have shown that limonenes were unstable and easily oxidized in the presence of oxygen [41]. The oxidation of limonenes finally produced many products such as limonene oxide, bornyl acetate, and others [42]. In this study, bornyl acetate was detected in rancid rather than normal samples, which strongly confirmed that *δ*-limonene was oxidized and degraded during the rancidity process. In addition, 2,3-butanediol, as high-value chemical, has great potential for diverse industries, including food, cosmetics, agriculture, and pharmaceutical areas [43]. (*R*,*R*)-2,3-Butanediol and (*R*,*S*)-2,3-butanediol were often detected in foods or TCMs as typical volatile compounds [44,45]. High-temperature and -humidity conditions caused them to volatilize and their content decreased significantly, which was consistent with the result that their content decreased in dried carrots after 50 d of storage [46].

Moreover, the ethanol concentration was 4.26% in normal samples and 0.75% in rancid samples which was caused by the normal samples being stored for 6 months in high-temperature and -humidity conditions. The result was consistent with the ethanol content decrease in kombucha during storage [47]. The high-temperature and -humidity conditions led to the growth and reproduction of bacteria in a confined space, some of which could metabolize ethanol to produce acetic acid [48,49,50]. Correspondingly, this led to a significant increase in acetic acid in rancid samples. Anethole, as an essential oil, has good potential for use as an alternative to synthetic fungicides for the preservation and storage of foods [51]. The content of it was decreased in this study, which was not favorable for the storage of ZSS. This was consistent with the result for the anethole content of sweet fennel fruits during aging [52]. *n*-Hexanol was positively correlated with pleasant odor and the overall valuation of the fruits, confirming that this lipid-related volatile compound was the most important for the aroma of foods such as “Ambrunés” sweet cherries [53]. Similarly, the descriptive words were sweet, spice, and herbal for estragole in previous literature [54]. The decrease in their content had negative impacts on flavor and odor of ZSS in this study. 2,3,5,6-Tetramethylpyrazine was an important compound responsible for the odor and possessed a unique organoleptic characteristic that could dramatically influence the sensory properties of foods and/or TCMs [55]. Its content was also decreased in rancid samples, which diluted the aromatic odor of ZSS. 2,2,4,6,6-Pentamethylheptane was often detected in foods such as tea and herbs [56,57], whose level became lower in rancid samples.

In terms of nine increased compounds, the relative content of acetic acid had the largest increase from 7.08 to 13.30%, followed by *n*-octanoic acid from 0.34 to 0.54%, and *n*-nonanoic acid from 0.17 to 0.27%. The largest increase in acetic acid content resulted in a stronger pungent odor from rancid samples. It was speculated that this was closely related to the degradation of ethanol [47]. Similarly, the content of butanoic acid and 3-methyl-butanoic acid was also increased, which was consistent with the result that they increased in rice wine during storage [56]. They were determined as odor-active compounds with unpleasant odors such as rancid, cheese-like, and fermented, which could be important contributors to flavor changes during fermentation and storage [58,59,60]. In addition, it was found that butanoic acid could be converted from lipids by intracellular enzymes in lactic acid bacteria [61]. Branched-chained amino acids such as valine, leucine, and isoleucine can be converted into branched-chained organic acids such as 2-methyl-butanoic acid and 3-methyl-butanoic acid by the fermentation and rancidity process during storage [62]. ZSS, as a seed foods or TCM, is rich in lipids and contains several amino acids [63]. High-temperature and -humidity conditions promoted the transformation of these compounds during storage.

Compared with the normal samples, as shown in Figure 4B, 19 unique compounds were detected and only one compound, *δ*-cadinene, was not detected in rancid samples. *δ*-Cadinene might be decomposed or transformed in the rancidity process. Nineteen compounds were produced in the rancidity process. Among them, the relative content of *β*-phellandrene was the highest at 10.46%, followed by *α*-pinene at 9.21%, and 3-carene at 7.74%. Related studies showed that *β*-phellandrene had a pleasant odor, *α*-pinene had a pine odor, 3-carene had a fruity odor, and *β*-myrcene had a pleasant and terpene odor [64,65]. In theory, the large increase in these olefin contents resulted in a stronger fragrance of rancid samples. On the one hand, the decreased level of the common volatile compound *δ*-limonene was more than the increased level of those unique compounds, so they failed to increase the fragrance of rancid samples. On the other hand, olefins generally have a higher odor threshold and do not contribute much to the overall odor [66].

Morever, the production of *o*-cymene and *β*-myrcene was proved to be closely related to lactic acid bacteria, and the increase in their content could promote lactic acid bacteria to become the dominant bacteria in ZSS during storage and an increase in butanoic acid content [46]. In addition, benzaldehyde is a product of oxidative degradation of fat. The relative content was not high in ZSS, but the threshold value of benzaldehyde was low, which contributed to the overall odor degree of rancid samples. Therefore, the increase in benzaldehyde still resulted in a stronger irritating odor of rancid samples [67]. The contents of both 5-ethyldihydro-2(3H)-furanone and 6-methyl-3,5-heptadiene-2-one were increased, but they did not smell and had little contribution to the overall odor of rancid ZSS samples. The content of 2-heptanol was also increased, which was consistent with previous studies on the storage of foods such as fragrant rice and roasted almonds [68,69].

Notably, the content of *δ*-limonene and anethole was decreased but that of *α*-pinene, *o*-cymene, *β*-myrcene, terpinen-4-ol, benzaldehyde, and bornyl acetate was increased in cooked mutton meatballs at a later stage of storage. As a result, the pleasant odor was gradually weakened and the unpleasant odor was more prominent [66]. This was consistent with the change in corresponding volatile compounds in this study, which further confirmed the credibility of the results.

The content of 11 organic acids in rancid samples was increased, including seven common and four unique compounds. They were a series of short-chain acids such as acetic, propanoic, butanoic, pentanoic, hexanoic, heptanoic, octanoic, and nonanoic acids. Their total relative content was from 13.63% in normal samples to 22.39% in rancid samples. These acids had some pungent and sweaty odors and could cause unpleasant odors when they were combined in rancid samples.

## 4. Conclusions

In this study, both temperature at 40 °C and relative humidity of 75% were set up to carry out the accelerated rancidity process for obtaining the rancid ZSS samples from normal ZSS samples over 6 months of storage time. It is worth noting that these storage conditions were not set at random, but were determined according to the parameters of the accelerated test and the drug stability investigation test, which could reflect the change process of substances and the stability of related physicochemical indicators [70,71,72]. In recent years, the condition of 40 °C and 75%RH was often used to evaluate the related changes and mechanisms during the storage of TCMs, foods, functional beverages [73], and cosmetics [74], such as pecan [75], Benjakul [76], *Salvia miltiorrhiza* [77], and *Piper lhotzkyanum* Kunth leaves [78].

After 6 months of storage, there was a significant difference between normal and rancid samples of ZSS. The most intuitive change is in traditional sensory properties, including the rancid samples producing a darker color, a bitter taste, and an irritating odor. This was consistent with the results that wild almond kernel, perilla seed, and beef samples became a darker color and had an unpleasant odor [79,80,81]. In addition, the physicochemical indicators such as moisture content, water- and alcohol-soluble extracts, and acid value were also detected. Among them, the acid value had a significant difference (*p* < 0.01), from 3.90 of normal ZSS to 18.68 mg/g of rancid ZSS. This was consistent with the results that the acid value had a significant increase in wild almond kernel from 0.07 to 0.83 mg/g after 30 d at 25 °C, large yellow croaker (*Larimichthys crocea*) from 2.02 to 7.62 mg/g after 120 d at 75%RH, peanut from 0.09 to 0.62 mg/g after 10 months at 35 °C, and green coffee beans from 0.65 to 1.28 mg/g after 20 days at 60 °C during storage link [81,82,83,84].

In order to further explore the changes in chemical composition from normal to rancid ZSS samples, HS-SPME-GC-MS technology was used to detect the volatile compounds. As a result, 39 volatile compounds were identified in ZSS samples, including 20 volatile compounds in normal samples and 38 volatile compounds in rancid samples. Nineteen common compounds were identified in normal and rancid samples. Among them, the content of 10 compounds such as *δ*-limonene, (*R*,*R*)-2,3-butanediol, and (*R*,*S*)-2,3-butanediol was decreased but that of nine compounds such as acetic acid, *n*-octanoic acid, and *n*-nonanoic acid was increased in rancid samples. Nineteen unique compounds such as *β*-phellandrene, *α*-pinene, and 3-carene were detected and only one compound, *δ*-cadinene, was not detected in rancid samples. It was speculated that *δ*-cadinene might be decomposed and 19 compounds were brought out in the rancidity process. It is worth noting that the total content of 11 organic acids such as acetic, propanoic, and butanoic acids in rancid samples was increased. This was consistent with the results that the content of some organic acids increased after the rancidity of high-quality extra virgin olive oils, French fries, poppy seeds, and sunflower seeds [85,86,87,88]. Among the many volatile compounds in the rancid samples, only some of them played an important role in contributing to the overall odor. Eight short-chain organic acids, acetic, propanoic, butanoic, pentanoic, hexanoic, heptanoic, octanoic, and nonanoic acids, were new products in rancid ZSS samples, and it was speculated that the production of a series of organic acids might be the material basis of irritating odors after normal ZSS became rancid. This is the first report that a series of short-chain organic acids have been found in a rancid substance.

For TCM or food processing enterprises, some raw materials of ZSS can be judged by traditional sensory properties whether the samples are rancid, and some are mixed with other raw materials, which requires the detection of physicochemical characteristics such as acid value and chemical components such as newly generated and increased compounds. For example, if a series of short-chain fatty acids or high levels of terpene compounds are detected in the sample, it means that the sample has become rancid and cannot be used as a raw material for production. Then, these rancid samples or mixed raw materials containing rancid samples can be eliminated, thus the quality of ZSS and related products can be improved.

In conclusion, the rancidity phenomenon of ZSS is a typical security problem in the storage link of TCMs and food industries. It is of great significance to quality control to find rancid materials. Our results showed that there was a significant difference in traditional sensory and physicochemical characteristics and chemical components between normal and rancid samples. These indicators could be used as an early warning or evaluation basis for judging the quality of medicinal and edible ZSS. In addition, this is the first comprehensive evaluation of the rancidity process of a medicinal and edible substance.

## Figures and Tables

**Table 1:** The traditional sensory properties of normal and rancid ZSS samples.

Sample	Color	Taste	Odor
Normal	Off-white	Slight bitter	Slightly fragrant
Rancid	Dark maroon	Bitter and sour	Irritating

**Table 2:** The physicochemical characteristics of normal and rancid ZSS samples (*n* = 6).

Sample	Moisture Content (%)	Water-Soluble Extract Content (%)	Alcohol-Soluble Extract Content (%)	pH Value	Electrical Conductivity (μs/cm)	Fatty Oil Content (%)	Acid Value (mg/g)
Normal	6.60 ± 0.06	26.35 ± 0.15	8.69 ± 0.04	6.09 ± 0.02	1229 ± 1	21.30 ± 0.30	3.90 ± 0.15
Rancid	7.64 ± 0.02 *	20.14 ± 0.05 *	5.64 ± 0.06 *	6.05 ± 0.01	1256 ± 3	16.04 ± 0.15 *	18.68 ± 0.47 **

* *p* < 0.05; ** *p* < 0.01.

**Table 3:** Volatile compounds of GC-MS analysis of normal and rancid ZSS samples.

No.	Rt (min)	Compound	MF	CAS	MW	Fragment (*m*/*z*)	Normal (%)	Rancid (%)
1	3.21	Ethanol	C_2_H_6_O	64-17-5	46	45/31/29/27	4.26 ± 0.12	0.75 ± 0.12 **
2	3.46	2,2,4,6,6-Pentamethylheptane	C_12_H_26_	13,475-82-6	170	155/112/99/57	4.93 ± 0.08	1.35 ± 0.07 *
3	4.46	*α*-Pinene	C_10_H_16_	2437-95-8	136	93/77/39/27	-	9.21 ± 0.18 **
4	7.59	3-Carene	C_10_H_16_	13,466-78-9	136	121/105/93/79	-	7.74 ± 0.12 **
5	8.20	*β*-Myrcene	C_10_H_16_	123-35-3	136	93/69/41/27	-	2.53 ± 0.06 **
6	9.24	*δ*-Limonene	C_10_H_16_	138-86-3	136	121/107/93/68	46.43 ± 0.61	15.97 ± 0.17 **
7	9.49	*β*-Phellandrene	C_10_H_16_	555-10-2	136	119/93/77/41/27	-	10.46 ± 0.09 ***
8	10.96	*γ*-Terpinene	C_10_H_16_	99-85-4	136	121/93/77/43	-	1.19 ± 0.06 **
9	11.96	*o*-Cymene	C_10_H_14_	527-84-4	134	119/91/77/65	-	4.92 ± 0.06 **
10	12.37	(+)-4-Carene	C_10_H_16_	5208-49-1	136	121/105/93/79	-	1.24 ± 0.06 **
11	14.05	2-Heptanol	C_7_H_16_O	543-49-7	116	98/83/55/45	-	0.68 ± 0.11 *
12	15.37	*n*-Hexanol	C_6_H_14_O	111-27-3	102	84/69/56/43	2.06 ± 0.14	1.54 ± 0.09
13	18.67	1-Methyl-4-(1-methylethenyl)-benzene	C_10_H_12_	1195-32-0	132	117/115/91/65	-	0.69 ± 0.07 **
14	19.48	Acetic acid	C_2_H_4_O_2_	64-19-7	60	45/43/29/15	7.08 ± 0.16	13.30 ± 0.34 ***
15	20.03	2,3,5,6-Tetramethylpyrazine	C_8_H_12_N_2_	1124-11-4	136	121/95/80/54/42	1.11 ± 0.08	1.02 ± 0.06
16	22.10	Benzaldehyde	C_7_H_6_O	100-52-7	106	105/77/51	-	1.06 ± 0.05 **
17	23.00	(*R*,*R*)-2,3-Butanediol	C_4_H_10_O_2_	24,347-58-8	90	75/57/45/29	13.22 ± 0.18	4.92 ± 0.05 ***
18	23.08	Propanoic acid	C_3_H_6_O_2_	79-09-4	74	73/57/45/28	-	0.84 ± 0.07 **
19	23.35	Linalool	C_10_H_18_O	78-70-6	154	136/121/93/71	0.32 ± 0.05	0.23 ± 0.05
20	24.24	Bornyl acetate	C_12_H_20_O_2_	76-49-3	196	154/136/121/95	-	0.46 ± 0.04 **
21	24.48	(*R*,*S*)-2,3-Butanediol	C_4_H_10_O_2_	5341-95-7	90	75/57/45/29	10.40 ± 0.09	5.40 ± 0.09 **
22	24.66	Humulene	C_15_H_24_	6753-98-6	204	147/121/93/80	-	0.32 ± 0.04 *
23	24.86	6-Methyl-3,5-heptadiene-2-one	C_8_H_12_O	1604-28-0	124	109/81/53/43/39	0.14 ± 0.02	0.27 ± 0.06
24	25.01	1,2-Propanediol	C_3_H_8_O_2_	57-55-6	76	61/45/43/31	-	0.36 ± 0.02 **
25	25.22	Terpinen-4-ol	C_10_H_18_O	562-74-3	154	136/121/93/71	-	2.30 ± 0.08 **
26	26.18	4-Hydroxy-butanoic acid	C_4_H_8_O_3_	591-81-1	104	96/86/56/42	-	0.82 ± 0.08 **
27	26.56	Butanoic acid	C_4_H_8_O_2_	107-92-6	88	73/60/41/27	0.23 ± 0.04	0.29 ± 0.04
28	27.87	Estragole	C_10_H_12_O	140-67-0	148	133/121/105/91	0.53 ± 0.09	0.31 ± 0.03 *
29	28.13	3-Methyl-butanoic acid	C_5_H_10_O_2_	503-74-2	102	87/69/60/43	1.57 ± 0.09	1.63 ± 0.12
30	28.91	5-Ethyldihydro-2(3H)-furanone	C_6_H_10_O_2_	695-06-7	114	85/70/56/42/29	-	0.71 ± 0.09 **
31	30.66	Pentanoic acid	C_5_H_10_O_2_	109-52-4	102	87/73/60/45	-	0.31 ± 0.06
32	30.86	*δ*-Cadinene	C_15_H_24_	483-76-1	204	189/161/134/119	0.21 ± 0.04	-
33	33.58	Anethole	C_10_H_12_O	104-46-1	148	133/117/105/91	2.22 ± 0.06	1.39 ± 0.08 **
34	34.50	Hexanoic acid	C_6_H_12_O_2_	142-62-1	116	99/87/73/60/41	3.98 ± 0.11	4.02 ± 0.17 **
35	36.53	Phenylethanol	C_8_H_10_O	60-12-8	122	103/91/65/51	0.27 ± 0.07	0.37 ± 0.07
36	38.14	Heptanoic acid	C_7_H_14_O_2_	111-14-8	130	101/87/73/60	0.26 ± 0.03	0.32 ± 0.03
37	41.86	*n*-Octanoic acid	C_8_H_16_O_2_	124-07-2	144	115/101/85/73	0.34 ± 0.04	0.54 ± 0.03 **
38	45.23	*n*-Nonanoic acid	C_9_H_18_O_2_	112-05-0	158	141/129/115/98	0.17 ± 0.03	0.27 ± 0.04 *
39	49.45	Benzoic acid	C_7_H_6_O_2_	65-85-0	122	105/77/51	-	0.05 ± 0.01

“MF” molecular formula, “MW” molecular weight, “-” not detected. Asterisks indicate significant differences between normal and rancid samples, * *p* < 0.05, ** *p* < 0.01, and *** *p* < 0.001.

## Data Availability

Data is contained within the article.
